# Using Cryopreserved *Plasmodium falciparum* Sporozoites in a Humanized Mouse Model to Study Early Malaria Infection Processes and Test Prophylactic Treatments

**DOI:** 10.3390/microorganisms11092209

**Published:** 2023-08-31

**Authors:** María-Belén Jiménez-Díaz, Jörg J. Möhrle, Iñigo Angulo-Barturen, Claudia Demarta-Gatsi

**Affiliations:** 1 The Art of Discovery, 48160 Derio, Basque Country, Spain; 2 Medicines for Malaria Venture, 1215 Geneva, Switzerland

**Keywords:** *Plasmodium*, FRG mouse model, cryopreserved parasites

## Abstract

In addition to vector control, long-lasting insecticidal nets and case management, the prevention of infection through vaccination and/or chemoprevention are playing an increasing role in the drive to eradicate malaria. These preventative approaches represent opportunities for improvement: new drugs may be discovered that target the early infectious stages of the *Plasmodium* parasite in the liver (rather than the symptomatic, abundant blood stage), and new, exciting vaccination technologies have recently been validated (using mRNA or novel adjuvants). Exploiting these possibilities requires the availability of humanized mouse models that support *P. falciparum* infection yet avoid the hazardous use of infectious mosquitoes. Here, we show that commercially available *P. falciparum* sporozoites and FRG mice carrying human hepatocytes and red blood cells faithfully recapitulate the early human malaria disease process, presenting an opportunity to use this model for the evaluation of prophylactic treatments with a novel mode of action.

## 1. Introduction

Malaria is still dominant among poverty-associated infectious diseases, causing extensive morbidity and mortality in resource-poor countries, with an estimated 241 million cases and 627,000 deaths in 2020 [1]. The eradication of such diseases poses significant challenges at the logistical, political and scientific levels, as well as priority considerations. An important pillar in the fight against malaria is the use of prophylactic treatments. These consist of seasonal chemoprevention, which covered approximately 40 million children in 2021 [2], mostly in the Sahel countries, and the emerging use of the RTS,S/AS01 vaccine [3]. Malaria chemoprevention, whether for children or travelers, is based on combinations of medicines incorporating a least one liver-stage-targeting drug with a blood-stage-targeting drug. Furthermore, the development of prophylactic treatments, focusing on drugs that target the initial, asymptomatic (liver, low-number) stages of *Plasmodium* parasite is critical in reducing the risk of the emergence of drug resistance, preventing disease progression by reducing clinical manifestation and severity and supporting malaria elimination efforts.

The RTS,S/AS01 (Mosquirix™) vaccine is only partially effective among children aged 5–17 months who receive four doses of it. However, the COVID-19 pandemic has significantly accelerated the clinical development of novel types of vaccination, namely the use of mRNA vaccines and novel saponin (‘Matrix M’) adjuvants [4]. Exploiting both these opportunities (targeting early stages of the parasite with drugs or using the new vaccination technologies) relies on the availability of suitable animal models. The deadliest human malaria parasite (*Plasmodium falciparum*) does not infect rodents, while in vitro cultures of liver or/and blood *Plasmodium* stages poorly translate to *in vivo* situations. There are *Plasmodium* species that infect rodents (*P. berghei*, *P. chabaudi* or *P. yoelii*), whilst the translation of such models to human disease has been questioned [5,6]. To overcome the limits of these surrogate models and to ensure the better translation of experimental findings, mouse–human chimeric models have been developed that allow the engraftment of human cells such as red blood cells and/or hepatocytes. Such animals do support infection with *P. falciparum* [7,8,9]. The establishment of a human liver-chimeric mouse model (Fah^−/−^/NOD/Rag2^−/−^/Il2Rg^−/−^, FRG KO huHep [9]) that can reliably be infected with *P. falciparum* sporozoites (SPZ) allows us to reproduce the *in vivo* biology of *P. falciparum* liver stages, including the replication of large schizonts [10]. This makes the model suitable for the study of new molecules capable of inhibiting the development of the parasite in its liver stages, supporting the development of new prophylactic therapies. Moreover, this model can be engrafted with human red blood cells (HuRBCs) and thus allow faithful replication of the complete parasite life cycle and the liver- to blood-stage transition of human *Plasmodia* species in a single animal model [11]—not only *P. falciparum* but also *P. vivax*, the second major malaria parasite, which is capable of delaying disease thanks to its hypnozoite stage in the liver [12]. Infection of such mice can be achieved using infectious mosquitoes; however, the breeding of infected mosquitos requires special biohazard laboratories. Moreover, the numbers of bites and injected SPZ vary. The use of chimeric mouse models could be significantly simplified with the use of cryopreserved *P. falciparum* SPZ [13,14]. Such SPZ are obtained from the salivary glands of infected mosquitoes, maintaining their viability and capacity to enter capillaries in the skin of the mammalian host and their passage with the circulation to the liver, infecting hepatocytes. The infection of chimeric mice with cryopreserved *P. falciparu*m SPZ and the replication of the live cycle in mice has previously not been achieved. Cryopreserved *P. berghei* SPZ infection has been tested *in vivo* [15]. Additionally, fresh SPZ have been used to infect FRG mice carrying human hepatocytes, but not red blood cells, resulting in incomplete infection [16]. Another approach uses liver-humanized FRGN KO (HLA-DR4.RagKO.IL2RgcKO.NOD) mice reconstituted with HuRBCs using an immune modulation protocol (clodronate-containing liposomes plus cyclophosphamide) to prevent the phagocytosis of infected HuRBCs (i-HuRBCs). However, this protocol is expensive and impractical [11]. Here, we show that commercially available cryopreserved *P. falciparum* SPZ(PfSPZ) from Sanaria can achieve productive liver infections in commercially available FRG mice (Yecuris Corp.) engrafted with human hepatocytes and HuRBCs, using a simplified protocol for HuRBC engraftment. More importantly, we provide evidence that this process faithfully mimics the natural early human disease process.

## 2. Results and Discussion

### 2.1. Successful Infection of Cryopreserved P. falciparum Sporozoites and Transition of Liver-Stage Parasites to Blood-Stage Parasites In Vivo

**Figure 1:**
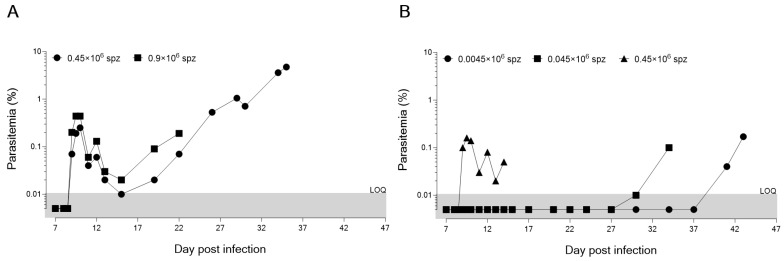
Parasitic growth curve (as measured by percentage of infected red blood cells) in humanized FRG mice engrafted with human hepatocytes and erythrocytes, after infection with cryopreserved *P. falciparum* SPZ at two (**A**) or three (**B**) inoculum doses. Two separate studies (**A**,**B**) were performed using one mouse (*n* = 1) per inoculum. The same batch was used for both studies.

An important part of the life cycle of the *Plasmodium* parasite in the mammalian host is the transition from the liver to the blood stage, when parasites emerging from the liver infect the first red blood cells. Previous studies have shown that FRG huHep mice infected with fresh *P. falciparum* SPZ support hepatocyte infection and liver-stage development, culminating in complete parasite maturation approximately 7 days after infection [16]. Here, we assessed the potential of cryopreserved *P. falciparum* NF135C10 (*Pf*NF135C10) SPZ to infect FRG KO huHep mice and developed this model further to allow exo-erythrocytic merozoite infection of human red blood cells and asexual blood-stage development. In two separate studies, mice were injected intravenously (i.v.) with different *Pf*NF135.C10 SPZ inoculates, 0.9 × 10^6^ and 0.45 × 10^6^ in the first study and 0.45 × 10^6^, 0.045 × 10^6^ and 0.0045 × 10^6^ in the second study (Figure 1A,B). Additionally, one mouse was infected with 0.2 × 10^6^ of SPZ for histological analysis.

The ability of cryopreserved SPZ to reach the liver and trigger malaria infection in the host was assessed daily by the quantification of i-HuRBCs. A fast and efficient flow cytometer-based protocol was used to count HuRBCs and the percentage of infected i-HuRBCs [17]. On day 9 post-infection, the percentage of non-infected HuRBCs in all the different inoculate groups was around 40–60%, providing a large pool of target cells for the emerging parasites. All animals developed blood-stage infection (Figure 1A,B). The only difference observed between the groups was the kinetics of i-HuRBC detection, which seemed to relate to the number of SPZ with which an animal was initially infected. Indeed, for the highest doses of inoculum (0.9 and 0.45 × 10^6^ SPZ), we observed similar kinetics of development in the blood (starting by day 9 p.i.). Furthermore, these inoculum doses produced similar kinetics of blood-stage establishment as those observed in the controlled human malaria infections (CHMIs) with cryopreserved SPZ,. Interestingly, quantitative PCR detected blood stage parasites in CHMIs, 9 to 12 days (13.0 days by microscopy) after challenge with 25 × 10^2^, 1 × 10^4^ and/or 25 × 10^3^ PfSPZ, with no differences in the pre-patent period between inocula, even though this delay ought to have been 2 days shorter in the 25 × 10^3^ PfSPZ group as compared to the 25 × 10^2^ PfSPZ group [18]. Similar results were also obtained using fresh SPZ in the DRAG KO mouse model, where *Plasmodium* infection led to a low but detectable blood-stage infection after 10–28 days [19]. When we tested lower doses (0.045 and 0.0045 × 10^6^ SPZ), the infection was detectable only at days 30 and 41 p.i., respectively (Figure 1B). Moreover, the low-inoculum groups produced erratic and delayed growth. This study allowed us to confirm the infectious capacity of cryopreserved SPZ and that 0.45 × 106 SPZ are sufficient to carry out assays of both liver- and blood-stage infection in the humanized FRG KO mouse model, with similar kinetics as seen in CHMI studies with cryopreserved SPZ [18,19,20].

### 2.2. Detection of Parasite Liver Infection by Microscopy Confirmed the Infectivity of Cryopreserved Sporozoites

**Figure 2:**
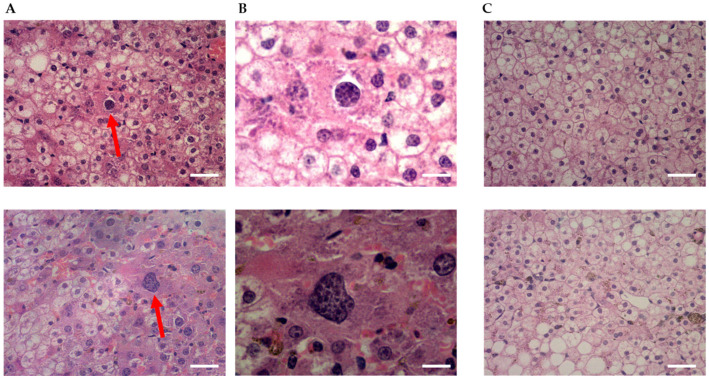
Qualitative assessment of parasitemia by haematoxylin-eosin staining of liver tissue. Shown are representative images of livers from (**A**,**B**) a mouse infected with 0.2 × 10^6^ cryopreserved SPZ of *P. falciparum* NF135.C10 on day 6 of the study (day 1: infection day) at 40× and 100× magnification, respectlvely, compared to (**C**) the control non-infected mouse at 40× magnitude. Red arrows show schizonts in the liver from the infected mouse (scale bar: (**A**,**C**) 25 μm and (**B**) 10 μm).

Histological evaluation of parasite infection in human hepatocytes was addressed to support earlier results (Figure 2). An FRG mouse was injected intravenously with *Pf*NF135C10 SPZ, sacrificed 5 days later, and liver tissue sections were prepared for analysis. This demonstrated that cryopreserved *P. falciparum* SPZ could infect and develop in the FRG huHep mice, similarly to what was observed in *in vitro* cultures. Multinucleated (>3 nuclei) spherical schizonts were detected, in agreement with what was previously observed by Vaughan and colleagues [16].

## 3. Conclusions

In this study, we demonstrated the ability of cryopreserved SPZ of *P. falciparum* NF135.C10 to infect and grow in FRG mice engrafted with human hepatocytes and human red blood cells similar to CHMIs. The results suggest that cryopreserved SPZ of *P. falciparum* NF135.C10 can be used as a tool in a standardized preclinical murine model of the liver stages of *P. falciparum*. Cryopreserved SPZ represent a reliable source of reagents for use in experiments of evaluation and allow greater control of logistical aspects than infections with fresh SPZ from mosquitoes. One explanation for the erratic growth seen with inocula smaller than 0.45 × 10^6^ is that, at a lower concentration, the number of infectious SPZ becomes a chance event where a small, final number of viable parasites ‘decide’ at what day infection becomes patent. The relationship between ‘infectious agents’ and patent infection is complex in natural hosts, let alone in reconstituted, immunocompromised animals. Nevertheless, our demonstration that the entire malaria infection process that follows the bite with a mosquito can be reproduced in mice allows for the testing of a variety of agents that act on the early disease process, be it vaccines, monoclonal antibodies or drugs with novel targets.

## 4. Materials and Methods

### 4.1. Ethics Statements

Studies with mice were performed at The Art of Discovery (TAD) and had been pre-approved by The Art of Discovery Institutional Animal Care and Use Committee (TAD-IACUC). This Committee is certified by the Biscay County Government (Bizkaiko Foru Aldundia, Basque Country, Spain) to evaluate animal research projects from Spanish institutions according to point 43.3 from Royal Decree 53/2013, from 1 February (BOE-A-2013-1337). All experiments were carried out in accordance with European Directive 2010/63/EU. The results from the animal experiments are reported following the ARRIVE guidelines (https://www.nc3rs.org.uk/arrive-guidelines, accessed on 4 October 2016), except for the disclosure of confidential business information. The human biological samples were sourced ethically, and their research use was in accordance with the terms of the informed consent.

### 4.2. Mice

The experiments were performed using 22–28 g female FRG^®^ KO mice on the NOD background (Yecuris Corporation, Tigard, OR, USA). These animals possess a knockout mutation of the Fah gene, resulting in a fumaryloacetate dehydrogenase deficiency that disrupts tyrosine metabolism, producing a hepatotoxic metabolite called fumarylacetate. Fumarylacetate causes murine hepatocyte damage and stimulates repopulation with human hepatocytes. This toxicity can be overcome by the oral administration of nitisinone (NTBC), which blocks the intracellular accumulation of this compound. Furthermore, the animals contain immune system-crippling mutations in the RAG-2 and the IL2Rg genes, with defects in macrophage regulation, NK activity and in T_reg_ cells. Once they reached the TAD facilities, the mice adapted for at least one week before entering the experimental procedures. The mice of the studies described herein were humanely sacrificed, by progressive CO_2_ asphyxiation and subsequent total blood exsanguination, when the criteria of end-point termination were met (e.g., >20% weight loss).

### 4.3. Parasites

The animals were infected with *Plasmodium falciparum* NF135.C10 sporozoites (SPZ). The sporozoites were purchased from Sanaria Inc. (Rockville, MD, USA).

## 5. Human Erythrocyte Engraftment

FRG KO huHep mice were engrafted with human red blood cells to have a minimum of 30–40% of human red blood cells circulating in peripheral blood. Mice received 1 mL of a 50% hematocrit erythrocyte suspension in RPMI1640 medium, 25% (*vol*/*vol*) decomplemented human serum, 3.1 mM hypoxanthine, by the intraperitoneal route [17]. The blood injections were daily administered from day 4 to day 7 of the study and then every two days until the end of the experiment.

## 6. Sporozoite Infection

SPZ were thawed following the protocol described by the supplier and diluted with room-temperature saline and injected into FRG KO huHep mice by the i.v. route (0.3 mL in the tail vein), using a single SPZ batch for both studies.

### 6.1. Blood-Stage Parasite Detection

The transition of the parasite from the liver to blood was followed by microscopy and flow cytometry. The detection of parasites by cytometry used the TER-119-Phycoerythrine monoclonal antibody (Miltenyi Biotec, Bergisch Gladbach, Germany) as a marker of murine red blood cells, and SYTO-16 (Invitrogen, Waltham, MA, USA), a non-selective fluorescent nucleic acid dye, to detect intraerythrocytic parasites phenotyped as TER-119^−^SYTO-16^+^ (non-mouse erythrocytes containing DNA), exactly as described in [17]. Briefly, serial 2 µL blood samples of peripheral blood from *P. falciparum*-infected mice were stained with TER-119-Phycoerythrine and SYTO-16 and then analyzed by flow cytometry (Attune NxT Acoustic Focusing Flow Cytometer). The routine limit of quantification was set to 0.01% (~10^6^ total red blood cells counted for detection of a minimum of 100 infected events as a statistically significant sample). The day of detection (DoD) was defined as the day that mice showed a blood parasite burden above the limit of quantification of 0.01%. The sporozoite quantification was set to zero when there were no detectable parasites in the peripheral blood by microscopy or by flow cytometry at the 0.01% limit of quantification by day 60 of the study. The DoD of these individuals was set at 60. Parasitemia was expressed as a percentage of infected red blood cells with respect to the total red blood cells in circulation and/or as the absolute concentration of circulating parasitized red blood cells. The detection by microscopy was carried out with peripheral blood samples taken throughout the experiment after being stained with Giemsa.

### 6.2. Liver-Stage Parasite Detection

The livers were removed at day 6 of the study, for histological analysis. Briefly, mice were euthanized with CO_2_ according to the protocol of TAD as approved by The Art of Discovery Institutional Animal Care and Use Committee (TAD-IACUC). The livers were extracted in sterile conditions and fixed with 4% neutral buffered formalin. Three days later, the livers were transferred to 50% ethanol for paraffin infiltration, and the infiltrated tissues were then embedded into wax blocks. Finally, slides with paraffin sections were stained with hematoxylin and eosin.

### 6.3. Software

Data analysis was performed using GraphPad Prism 7.0 (GraphPad Software) and Excel 2016 (Microsoft).

## Data Availability

The datasets analyzed during the current study are available from the corresponding author on request and with permission from Medicines for Malaria Venture.
